# Biocatalytic Amplification of UV Signal in Capillary Electrophoresis of MicroRNA

**DOI:** 10.3390/ijms21010051

**Published:** 2019-12-19

**Authors:** Ruibin Hu, Yi Chen

**Affiliations:** 1 Key Laboratory of Analytical Chemistry for Living Biosystems, Institute of Chemistry, Chinese Academy of Sciences, Beijing 100190, China; huruibin66@iccas.ac.cn; 2 University of Chinese Academy of Sciences, Beijing 100049, China; 3 Beijing National Laboratory for Molecular Science, Beijing 100190, China

**Keywords:** microRNA catalysis, DNA hybridization, UV signal non-PCR amplification, non-gel capillary electrophoresis, determination

## Abstract

MicroRNAs (miRNAs) are new potential biomarkers for early diagnosis and classification of cancer. This study is the first attempt to use biocatalytic amplification reactions combined with capillary electrophoresis to detect multiple miRNAs simultaneously. In this way, miRNAs, as catalysts, can catalyze two single strands of DNA to form double-strand DNA. Feasibility was demonstrated by non-gel capillary electrophoresis coupled with UV detection (NGCE-UV). The detection limit was improved down to 1.0 nM, having ca. 10^3^-fold improvement. This method has a good linear range of between 3.0 nM and 300 nM, with R^2^ at 0.99, recovery at 88–115%, and peak area precision at 1–12.7%. Using three target miRNAs as a model can achieve the baseline separation and good selectivity. The proposed biocatalysis coupled with a capillary electrophoresis-based method is simple, rapid, multiplexed, and cost-effective, making it potentially applicable for simultaneous, large-scale screening for other nucleic acids biomarkers and related research.

## 1. Introduction

MicroRNAs (miRNAs), which are composed of approximately 20 nucleotides, are a huge class of small endogenous RNAs. More than 2800 miRNAs have been found, 50% of which are in cancer-related chromosomal regions and fragile sites [1]. Their abnormal levels are closely associated with the occurrence and development of multiple tumors [2,3] and other diseases [4,5,6]. For instance, Wu et al. confirmed high expression level of miRNA-21 and miRNA-29a in the serum of breast cancer patients using a SOLiD sequencing-based technique [7]. Zhu et al. reported that the content of serum miRNA-155 was higher in patients with progesterone receptor (PR)-positive tumors than in the PR-negative group [8]. Zhu et al. confirmed that 40 miRNAs are significantly dysregulated in type 2 diabetes by meta-analysis [9]. Many miRNAs have been shown to be stable in biological fluids, including tears, saliva, semen, urine, blood, and breast milk [10,11,12,13,14]. Presently, it is known that a single miRNA may appear in a variety of diseases, while the progression and development of a single disease may also be associated with multiple miRNAs [15,16,17]. Hence, they are potentially new biomarkers.

In order to improve the early diagnosis and classification of diseases, it is necessary to accurately and rapidly analyze multiple miRNAs in clinical samples. Various methods have been tried, ranging from Northern blotting [18,19] to quantitative reverse transcription polymerase chain reaction (qRT-PCR) [20,21]. Northern blotting is an early classical method for miRNA analysis, but it needs a large amount of samples and labor-intensive steps, which limited its broad applications [22]. With qRT-PCR, it is easy to generate amplification-related errors and it requires expensive equipment and consumables [23]. In theory, capillary electrophoresis (CE) is a better alternative, based on its unique features such as simplicity, rapidity, nanoliter consumption of samples, and ultra-high power of separation (i.e., high theoretic plate count or short theoretic plate height). Pioneer works exploring CE of miRNAs have been carried out. Jiang et al. designed specific single-strand DNAs (ssDNAs) with different lengths as a tandem adenosine-tailed bridge to hybridize the target miRNAs [24]. Ban et al. labeled different fluorescent groups at the end of DNA probes in their dual LIF detection, achieving simultaneous detection of three kinds of miRNA_S_ [25]. Wegman et al. demonstrated that CE can be an effective tool for simultaneously detecting multiple miRNAs by utilizing different drag tags of DNA probes, modified with fluorescent substances [26,27]. The “drag tags” are herein some small molecules (i.e., amino acids or small peptides) labeled on target analytes to change their size-to-charge ratios for better resolution. Li et al. combined rolling circle amplification with CE-LIF for detecting small RNA [28]. In these methods, fluorescent markers are essential, limiting their wide application.

Considering that CE-UV is convenient to use and universally applicable for determining miRNAs without fluorophore, we set out to make it usable in miRNA analysis. Our idea was to largely improve the UV detection signals through various cyclic amplification strategies, with a particular focus on miRNA-associated biochemical catalytic reactions. Following a period of inspections, a non-PCR biochemical amplification reaction caught our attention. This is a type of DNA hybridization reaction catalyzed by miRNA [29], which has been intensively studied in our CE-UV of miRNA.

In this study, a miRNA-catalyzed cyclic hybridization of ssDNAs into dsDNAs was introduced to the CE-UV analysis of miRNAs and discussed. Signals were acquired from the dsDNA rather than miRNAs themselves. To size-separate the nucleic acids with high stability, non-gel capillary electrophoresis with UV detection (NGCE-UV) was adopted, although the electropherogram was plotted against migration charge *Q* (to be presented in the following sections) rather than migration time *t*. The feasibility of this innovative method was demonstrated via a fine design of DNA sequence, as well as optimization of DNA hybridization and CE conditions. The combination of biocatalysis and the capillary electrophoresis method has great potential for screening other nucleic acid biomarkers for the early diagnosis of various diseases and related research.

## 2. Results and Discussion

### 2.1. Principle to Amplify the UV Signals of MiRNAs

**Figure 1:**
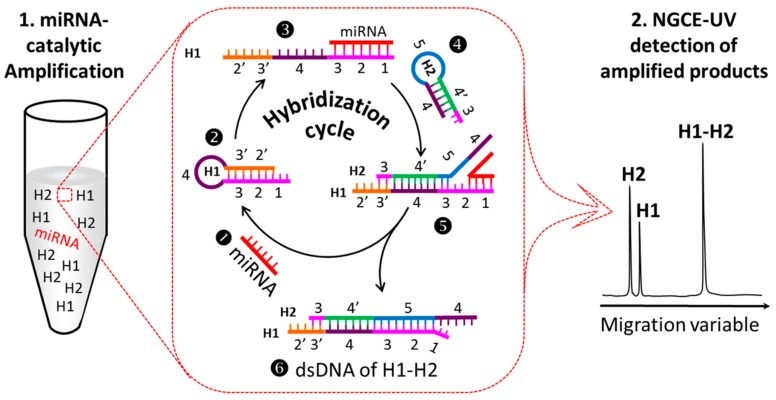
Principle for non-gel capillary electrophoresis with UV detection (NGCE-UV) detection of a specific miRNA through its initiation and catalysis of a cyclic hybridization reaction between two sequence-complementary single hairpin DNAs, H1 and H2.

A route map for non-PCR amplification of the NGCE-UV signals in the analysis of miRNAs is illustrated in Figure 1. Two apparent steps are included. The first step is to amplify the target products by miRNA-catalyzed cyclic hybridization of two complementary ssDNAs in a vial (left in Figure 1), and the second is to separate and detect the products by NGCE-UV. The procedure is clear but the involved reaction mechanism is complicated, which is can be further explained as follows.

The detection of target miRNAs is transferred to detect the dsDNA, which is formed and amplified by the miRNA-catalyzed cyclic hybridization of two complementary ssDNAs, named H1 and H2. The two complementary hairpin ssDNAs cannot hybridize automatically as they mix. However, if one of the ssDNA, such as H1, has a section of sequence at one end that also complements the target miRNA, the stem of the hairpin will be opened when the target miRNA appears (❶ to❸ in Figure 1). The opened end of H1 is now free and will have a large chance to capture its complementary ssDNA H2, to proceed their hybridization reaction (❹ to ❺ in Figure 1). As hybridization completes (❻ in Figure 1), the bound miRNA will be squeezed out of the H1 end as H1–H2 is stronger than H1–miRNA. The released miRNA will then search for another H1 to hybridize, initiating a cyclic hybridization of H1 with H2. In this way, a large amount of H1–H2 complexes will be formed with only a minute amount of catalyst miRNA. Due to the high specificity of the designed hybridization, we can indirectly detect the minute miRNA by separation and detection a large number of H1–H2 complexes.

**Figure 2:**
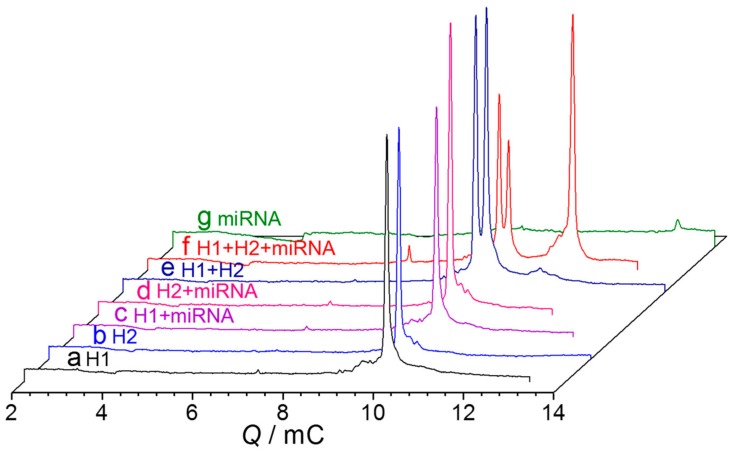
*Q*-electropherogram for NGCE-UV of miRNA-155 and its catalyzed hybridization products of ssDNA H1 and H2. Capillary: 100 μm I.D. ×20 (effective)/27 cm; coolant temperature: 25 °C; Running buffer: 100 mM Tris, 100 mM boric acid, 2 mM EDTA, and 6% PVP at pH 8.3; injection: −4 kV for 8 s; electric field: −400 V/cm; datum rate: 5 Hz; sample: (**a**) 4.0 μM H1; (**b**) 4.0 μM H2; (**c**) 4.0 μM H2 and 200 nM miRNA-155; (**d**) 4.0 μM H1 and 200 nM miRNA-155; (**e**) 4.0 μM H1 and 4.0 μM H2; (**f**) 200 nM miRNA-155, 4.0 μM H1, and 4.0 μM H2, at 37 °C for 2.5 h; and (**g**) 1.0 μM miRNA-155.

The cyclic hybridization reaction can produce sufficient H1–H2 complexes for NGCE separation and direct UV detection. As Figure 2 shows, the H1–H2 complexes are well separated from H1, H2, and miRNA, yielding a very high peak of UV absorption compared with miRNA. As controls, the isolated injection of individual H1, H2, or miRNA produced no peak(s) at the position of H1–H2 complexes, except for the mixture of H1 and H2, which produced a small peak near the H1–H2 peak (Figure 2e). As expected, the detection of H1–H2 hybrids could largely improve the detection signal, with detection limit reduced from ca. 1.0 μM miRNA to 1.0 nM, having an improvement of ca. 10^3^-fold. This not only makes full use of the characteristics of UV absorption of the nucleic acid itself, but is also convenient and the low-cost.

**Figure 3:**
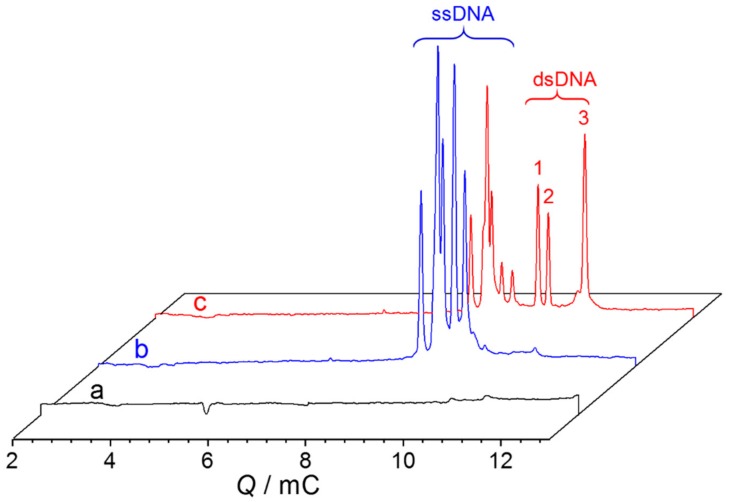
*Q*-electropherogram for NGCE-UV of (**a**) three miRNAs, (**b**) three pairs of H1 and H2 without the addition of any miRNA, and (**c**) three miRNAs incubated with their related pairs of H1 and H2. Sample: (**a**) miRNA-29a, miRNA-21, and miRNA-155 mixed at 200 nM each; (**b**) mixture of the three pairs of H1 and H2 at 4.0 μM each; (**c**) mixture of miRNA-29a, miRNA-21, and miRNA-155 at 200 nM each, and their H1 and H2 probes at 4.0 μM each. Other conditions as in Figure 2. Peak identity: 1: H1–H2 for miRNA-29a, 2: H1–H2 for miRNA-21, 3: H1–H2 for miRNA-155.

To validate the applicability of the proposed method for the simultaneous determination of multiple miRNAs, three specific miRNAs were tested as a model. While miRNAs are detectable at high concentration, they are not at a concentration below 200 nM (Figure 3a). Three expected peaks of H1–H2 hybrids appeared when the target miRNAs, along with their corresponding H1 and H2, were mixed in a same reaction system at the same time (Figure 3c). Impressively, the three dsDNAs of (H1–H2)s were baseline-separated. This can be used for quantitative analysis of multiple miRNAs. There is, herein, a situation that needs to be explained—the sequence size difference between H1_29a_ and H2_21_ is small, so they cannot be separated well, but this does not affect the quantitative analysis (Figure 3b).

### 2.2. Critical Parameters Associated with the Method

To improve the cyclic amplification signal, it is essential to optimize the sequence design of H1 and H2, the cyclic hybridization reaction, and the separation conditions.

#### 2.2.1. Design and Optimization of H1 and H2 Sequences

It is critical to design a pair of H1 and H2 for a specific miRNA well. For convenience, we divided H1 into 6 regions marked as 1, 2, 3, 4, 3′, and 2′, respectively (❷ in Figure 1). Regions 1, 2, and 3 are totally complementary to the target miRNA. Region 1 serves as a hook for capturing target miRNA when H1 is in a hairpin state (❶, ❷, and ❸ in Figure 1). The length of region 1 is approximately eight bases, which are decided by its ability to hook the target miRNA and are judged by the initiation rate of the hybridization reaction. Regions 2′ and 3′ are complementary to 2 and 3, respectively, of which region 3′ serves as a second hook to capture H2 (❹ and ❺ in Figure 1). Through the use of this design, the expected hybridization was initiated by the target miRNA (❶, ❷, and ❸ in Figure 1) to form the H1–H2 complex (❺ in Figure 1). The miRNA will finally squeeze out from H1 if region 5 is complementary to 3 and 2. The released miRNA (❶ in Figure 1) can then start a new cycle of hybridization reaction.

Based on the sequence of H1, the H2 sequence should have regions 4 and 5, followed by region 4′, to ensure its perfect hybridization with H1. Region 5 is determined by the target miRNA, where only region 4 or region 4′ is designable, which determines the stem strength of the hairpin H2 and is associated with the loop design of H1. A set of hairpin structures, H1 and H2, were carefully designed for target miRNA-21, miRNA-29a, and miRNA-155 based on the above, as well as the principle of the enzyme-free amplification system [30,31]. The detailed sequences were collected in Table S1.

#### 2.2.2. Reaction Conditions

**Figure 4:**
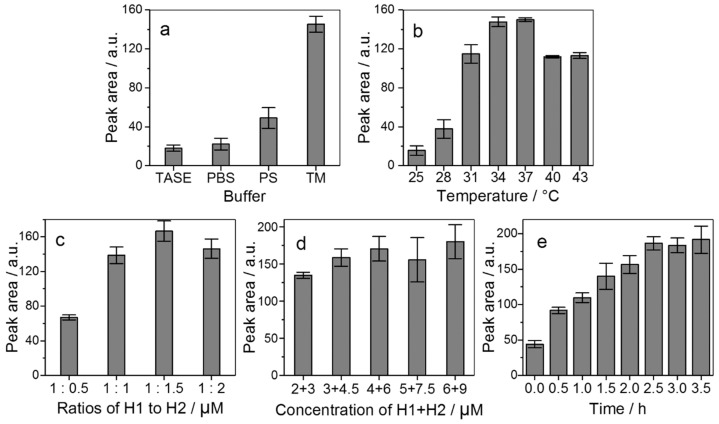
Variations of UV absorption peak with (**a**) hybridization buffer, (**b**) reaction temperature, (**c**) ratios of H1 to H2, (**d**) total concentration of H1 + H2, and (**e**) incubation time, with error bars calculated over triplicate. TASE: 50 mM Tris-acetate, 50 mM NaCl, and 10 mM EDTA at pH 8.0; PBS: 10 mM Na_2_HPO_4_, 10 mM NaH_2_PO_4_, and 137 mM NaCl at pH 7.4; PS: 10 mM Na_2_HPO_4_ and 2 mM NaCl at pH 7.4; and TM: 10 mM Tris and 2 mM MgCl_2_ at pH 7.4. Testing sample: 50 nM miRNA-21. Other capillary electrophoresis (CE) conditions as in Figure 2.

Cyclic hybridization conditions were expected to impact largely on detection performance. Reaction buffer was first checked with 50 nM miRNA-21 as a testing sample to catalyze the hybridization of H1 with H2. Several commonly used buffers were tested, including TASE (50 mM Tris-acetate, 50 mM NaCl, and 10 mM EDTA at pH 8.0), PBS (10 mM Na_2_HPO_4_, 10 mM NaH_2_PO_4_, and 137 mM NaCl at pH 7.4), PS (10 mM Na_2_HPO_4_ and 2 mM NaCl at pH 7.4), and TM (10 mM Tris and 2 mM MgCl_2_ at pH 7.4). The results (Figure 4a) show that TASE produces the lowest peaks of H1–H2 complexes, PBS and PS give a bit of a higher yield, although still much less than TM, which yields the highest peaks. TM was thus chosen as the reaction buffer and used throughout the study.

The hybridization reaction temperature should also be optimized as it affects the reaction rate and equilibrium. With the same testing miRNA and the suggested TM reaction buffer, the miRNA-catalyzed DNA hybridization reaction was found to work well between 25 °C to 43 °C, much similar to an enzymatic reaction. The optimal temperature is at 34 or 37 °C (Figure 4b), while reaction at 37 °C is adopted for potential physiological studies.

The concentrations of the reactants H1 and H2 are typically overloaded by approximately 20-fold over the target miRNA(s) to produce sufficient H1–H2 hybrids. It was unexpected that the ratio of H1 to H2 did not remain at 1:1, but was 1:1.5 instead (Figure 4c). Detection sensitivity increased with total concentration of H1 + H2, almost reaching a plateau at 4 + 6 μM (Figure 4d).

Under these optimized conditions, the incubation took about 2.5 h to reach a plateau or equilibrium (Figure 4e). It appears reasonable that the miRNA cannot catalyze the reaction indefinitely as it may lose out gradually due to breakage, nonspecific adsorption to reaction container, and possibly to some H1–H2.

#### 2.2.3. NGCE-UV Conditions

Three aspects were considered when performing NGCE-UV of dsDNA samples. The first was to improve its peak stability, which was resolved by plotting *Q*-electropherogram instead of *t*. The second was to select an optimal absorption UV wavelength, which was achieved by simply checking the UV absorption spectra, giving the maximal wavelength at 260 nm in general (Figure S1). In this study, 254 nm was chosen to detect the peaks, given that it is readily equipped on the instrument.

**Figure 5:**
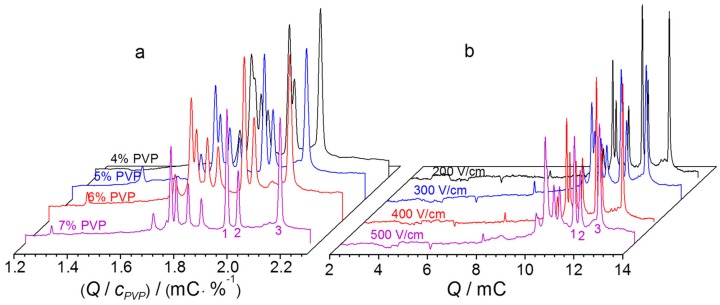
Impact of (**a**) PVP and (**b**) voltage on NGCE resolution. Running buffer: 100 mM Tris, 100 mM boric acid, 2 mM EDTA, and (**a**) 4–7% or (**b**) 6% PVP, all at pH 8.3; electric field: (**a**) 400 V/cm or (**b**) from 200 V/cm to 500 V/cm; sample: miRNA-21, miRNA-29a, and miRNA-155 (200 nM each) mixed with their corresponding pairs of H1 and H2 (4 μM each), and incubated at 37 °C for 2.5 h. Other conditions as in Figure 2. Peak identity: 1: miRNA-29a-induced H1–H2; 2: miRNA-21-induced H1–H2; 3: miRNA-155-induced H1–H2.

The third aspect was to have a better resolution for differentiating the target peaks from others. The application of polymer as sieving matrix in DNA separation has been widely reported. To achieve size separation, we adopted PVP as a non-gel sieving matrix as its aqueous solution is not very viscous but has an excellent self-coating ability, which suppresses the electroosmotic flow [32,33,34]. A bare fused-silica capillary can therefore be used directly for separation. With three mixed miRNA-21, miRNA-29a, and miRNA-155 as a test sample, we found that NGCE-UV should be carried out at a field ≥200 V/cm in a buffer containing ≥5% PVP to completely separate the three pairs of H1–H2 complexes from their reactants (Figure 5). In this study, we adopted the conditions of 6% PVP at 400 V/cm to have baseline resolution and kept the whole separation time in 6 min.

### 2.3. Method Validation

To investigate the feasibility of the strategy for quantitative target miRNAs detection, we measured H1–H2 complexes’ signal change responses at different concentrations of target miRNAs under the optimal experimental conditions. Linear range for miRNA-21, miRNA-29a, and miRNA-155 were obtained between 3.0 nM and 300 nM (Figure S2), with all linear coefficients above 0.99. Precision measured by peak area was below 9.7% (RSD, relative standard deviation) for intraday assays and below 12.7% for interday. Recovery was 88–115%. The detailed results are shown in Table 1.

**Figure 6:**
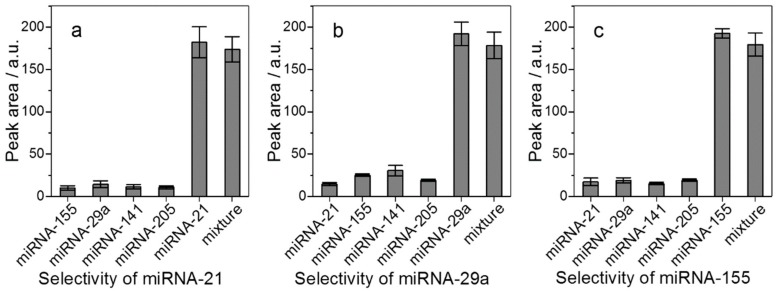
Selectivity test of the proposed method. (**a**) The target miRNA is miRNA-21, (**b**) is miRNA-29a, and (**c**) is miRNA-155. Error bar calculated over triplicate. Other conditions as in Figure 2.

Selectivity was evaluated by the addition of two nontargets of miRNA-141 and miRNA-205 into the reaction mixture of miRNA-21, miRNA-29a, and miRNA-155. Under the same experimental conditions, the peak data (Figure 6) indicated that the products of H1–H2 complexes were not significantly different when target miRNAs or a mixture of five miRNAs were presented in the same reaction system. Furthermore, the results of H1_21_ single base mismatch and various fragments of RNA were also good (Figure S3). These results suggest that the method has a good selectivity for target miRNA detection.

In order to demonstrate the potential application of this method in a physiological condition, human sera samples were spiked with three target miRNAs—miRNA-21, miRNA-29a, and miRNA-155—at three levels of concentration: low at 10 nM, medium at 50 nM, and high at 200 nM. They were then subjected to NGCE-UV detection. The measured recoveries were between 85% and 112%, and so were suitable for quantification (Table 2). This implies that the method can well overcome the interference from the very complex substances in sera, which include a variety of plasma proteins, nucleases, peptides, fats, hormones, inorganic substances, and so on. This provides a basis for further development of a method for determining miRNA in real samples.

## 3. Materials and Methods

### 3.1. Materials

All miRNA-correlated hairpin DNAs (each set of H1 and H2) and miRNAs of HPLC grade were synthesized by Shanghai Sangon Biotechnology Co., Ltd. (Shanghai, China). Their sequences were laboratory-designed and listed in Table S1. Polyvinylpyrrolidone (PVP) (average *M_W_* 1,300,000 g/mol) and other reagents were purchased from Sigma-Aldrich (St. Louis, MO, USA). Water used in this study was purified to resistance ≥18.2 MΩ/cm by Millipore water purification system (Bedford, MA, USA). Unless otherwise specified, all chemicals and reagents were of analytical grade.

### 3.2. Preparation of dsDNAs by MiRNA-Catalyzed Cyclic Hybridization

#### 3.2.1. Preparation of Hairpin DNAs

All oligonucleotides were dissolved in 1 × TE buffer (10 mM Tris and 1 mM EDTA, pH 8.0) solution, denatured at 95 °C for 3 min and then cooled down to room temperature for 1 h to ensure their formation of perfect hairpin structure. They were then stored at 4 °C to be used later. Synthesized miRNAs were separately prepared in diethyl pyrocarbonate (DEPC)-treated water and stored at −20 °C for later use.

#### 3.2.2. Hybridization Reaction

A pair of hairpin DNAs, H1 and H2, were mixed together with their target miRNA in a reaction buffer composed of 10 mM Tris and 2 mM MgCl_2_ (TM) at pH 7.4. The mixed solution was then incubated at 37 °C for 2.5 h and subjected to separation by CE-UV or stored at 4 °C if necessary. During the processes, the miRNAs were stable (Figure S4).

### 3.3. Capillary Electrophoresis

All separation experiments were performed on P/ACE 5500 capillary electrophoresis system (Beckman–Coulter, Fullerton, CA, USA) equipped with a UV detector and a capillary of 20 (effective)/27 cm (total) × 100 μm I.D./365 μm O.D. from Yongnian optical fibers (Hebei, China). A new capillary was flushed with 0.1 M NaOH for 10 min, 0.1 M HCl for 5 min, and water for 5 min. Between each run, the capillary was sequentially flushed with water, followed by 0.1 M HCl and water for 0.5 min each. Prior to the injection, the capillary was rinsed with a running buffer (100 mM Tris, 100 mM boric acid, 2 mM EDTA, and 6% PVP, at pH 8.3) for 2 min. The sample was injected at −4 kV for 8 s, separated at −400 V/cm and 25 °C (coolant), and UV-detected at 254 nm. Data were acquired at 5 Hz and further treated by a laboratory-edited software to calculate the migration charge *Q*. Where needed, Origin Pro 2017 software was also adopted to analyze the acquired CE data.

CE and its submodel of NGCE tend to be subjected to irreproducible and unrepeatable issues. This greatly affects their identification and quantification performance. To overcome these issues, or to improve the stability of peak position, Lee et al. proposed, as early as in 1991, to replace the migration time with migration indices [35], while Zhao et al. suggested, in 2004, using CE with an electric flux [36] as a measurable parameter. A bit later, in the deduction of diffusion spectrometry, our group adopted a migration charge, that is, *Q*, to perform voltage-independent CE [37]. A brief theoretical deduction is given below, which starts from the common CE principle:

(1) $$\upsilon =\upsilon_{ep}+\upsilon_{eo}=(\mu_{ep}+\mu_{eo})\;E=\frac{2}{3Sk}\frac{\varepsilon }{\eta }(\zeta_{a}+1.5\;\zeta_{c})I$$

where *υ* is migration velocity and *μ* is mobility (that is, velocity per unit of electric field strength *E*, which is equal to current *I* over the capillary cross sectional area *S* and buffer conductivity *k*). The subscript *ep* and *eo* indicate the electrophoresis and electroosmosis, respectively, while *ε* is the dielectric constant of the running buffer, *η* is its viscosity, *ζ_a_* is the electrokinetic potential of ion species, and *ζ_c_* is the zeta potential at the shearing interface away the capillary inner wall. This equation can be integrated by migration time from 0 to *t_R_* at *υ* = *dL*/*dt*:

(2) $$\overset{L_{R}}{\underset{0}{\int }}dL=\overset{t_{R}}{\underset{0}{\int }}\frac{2\varepsilon }{3S\eta k}\left(\zeta_{a}+1.5\zeta_{c}\right)Idt$$

where *L_R_* is the migration distance. If the variations of *ε*, *η*, *k*, and *ζ* with time are neglected, the integral gives:

(3) $$\left\{\begin{array}{c} \;Q=\overset{t_{R}}{\underset{0}{\int }}Idt=1.5SL_{R}\frac{\eta k}{\varepsilon \left(\zeta_{a}+1.5\zeta_{c}\right)}=1.5q_{R}\frac{\eta k}{\varepsilon \left(\zeta_{a}+1.5\zeta_{c}\right)}\\ q_{R}=SL_{R} \end{array}\right.$$

where *q_R_* denotes a migration volume of a species, being fixed for a given CE system. The corresponding migration charge *Q* can be measured in real time through the measurement of timely current *I* and integration with *t*. Clearly, *Q* is independent of the electrophoretic voltage or current applied. It can thus eliminate the influence of voltage or current on peak position if an electropherogram is plotted against *Q* rather than time *t*. This is the core reason why we adopted here a rarely used but helpful model of *Q*-NGCE to perform miRNA analysis. By using a P/ACE system, it was possible to record the current signals in real time, and we could thus calculate *Q* and plot *Q*-electropherogram in addition to *t*-one. The calculation was achieved with a short program and edited using LabView or by postanalysis with OrignPro or Excel.

## 4. Conclusions

In summary, the combination of biocatalysis and capillary electrophoresis for signal amplification of miRNA has been successfully developed as a method in which the miRNA acts as the catalyst to trigger a cyclic hybridization reaction of two miRNA-associated ssDNAs. No enzyme is needed to assist the reaction. This study demonstrated that this method can improve ca. 10^3^-fold when compared to the direct CE-UV of miRNA and that it has a good selectivity and anti-interference ability. Moreover, this proposed approach can not only detect multiple miRNAs simultaneously, but also does not need to label any fluorescent substrates. This greatly improves its application scope. In addition, this simple, isothermal cyclic amplification method avoids tedious steps and temperature cycling compared with PCR-based methods. It is realized by substituting the target specific sequence, while the two-layer biochemical amplification strategy can also be easily extended for determining other analytes. Therefore, the developed method can be easily realized in all laboratories and has a large space to screen other nucleic acid biomarkers and analytes for early diagnosis of diseases and biomedical-related research.

## Figures and Tables

**Table 1:** Linearity, precision, and recovery of target miRNAs measured by the proposed approach.

Targets	Calibration Curve (R^2^)	Low Concentration (10 nM, *n* = 3)			Medium Concentration (50 nM, *n* = 3)			High Concentration (200 nM, *n* = 3)		
		Intraday RSD (%)	Interday RSD (%)	Intraday Recovery (%)	Intraday RSD (%)	Interday RSD (%)	Intraday Recovery (%)	Intraday RSD (%)	Interday RSD (%)	Intraday Recovery (%)
miRNA-21	Y = 2.79*c* + 24.74 (0.9976)	2.31	9.11	88.4	3.03	2.07	99.3	4.45	1.67	95.1
miRNA-29a	Y = 2.72*c* + 34.05 (0.9948)	9.66	12.61	95.0	2.50	1.21	108.6	1.39	1.97	89.9
miRNA-155	Y = 2.97*c* + 38.53 (0.9962)	9.24	3.66	115.4	1.05	2.09	110.9	5.66	4.36	97.1

**Table 2:** Analysis results of target miRNA spiked in human sera samples by this method.

Targets	Added Concentration (nM)	Measured Concentration (nM)	RSD (%) (*n* = 3)	Recovery (%) (*n* = 3)
miRNA-21	10	10.2 ± 0.5	4.79	101.8
	50	52 ± 2	3.55	104.1
	200	210 ± 4	1.95	105.2
miRNA-29a	10	8.5 ± 0.7	8.17	85.1
	50	56 ± 3	4.8	112.8
	200	215 ± 12	5.37	93
miRNA-155	10	10.4 ± 0.7	6.7	104.3
	50	53 ± 5	9.63	105.8
	200	204 ± 4	1.87	101.9

## Supplementary Materials

Supplementary materials can be found at https://www.mdpi.com/1422-0067/21/1/51/s1.

## Abbreviations

CE	capillary electrophoresis
DEPC	diethyl pyrocarbonate
LIF	laser-induced fluorescence
miRNAs	microRNAs
NGCE-UV	non-gel capillary electrophoresis coupled with UV detection
PVP	polyvinylpyrrolidone
qRT-PCR	quantitative reverse transcription polymerase chain reaction
ssDNAs	single-strand DNAs
dsDNAs	double-strand DNAs
