# Supplementary material for: Biocatalytic Amplification of UV Signal in Capillary Electrophoresis of MicroRNA
Source: Int J Mol Sci. 2019 Dec 19;21(1):51. doi: 10.3390/ijms21010051 (PMC6981575; doi:10.3390/ijms21010051)
Supplement: Supplementary file 1 [file ijms-21-00051-s001.pdf]

Supplementary materials:

Biocatalytic Amplification of UV Signal in Capillary Electrophoresis of MicroRNA

Ruibin Hu [1,2] and Yi Chen [1,2,3,*]

[1] Key Laboratory of Analytical Chemistry for Living Biosystems, Institute of Chemistry, Chinese Academy of Sciences, Beijing 100190, China; huruibin66@iccas.ac.cn
[2] University of Chinese Academy of Sciences, Beijing 100049, China
[3] Beijing National Laboratory for Molecular Science, Beijing 100190, China
* Correspondence: chenyi@iccas.ac.cn

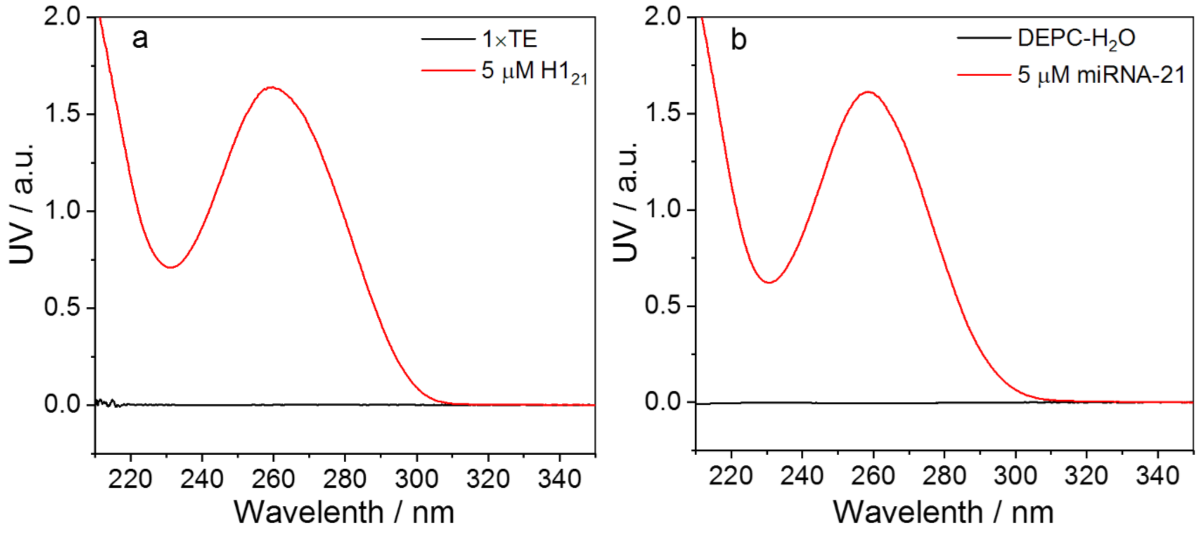

Figure S1. DNA ultraviolet absorption spectrum. (a) $H1_{21}$ were dissolved in 1×TE buffer (10 mM Tris and 1 mM EDTA, pH 8.0) solution. (b) miRNA-21 were dissolved in diethyl pyrocarbonate (DEPC) treated water.

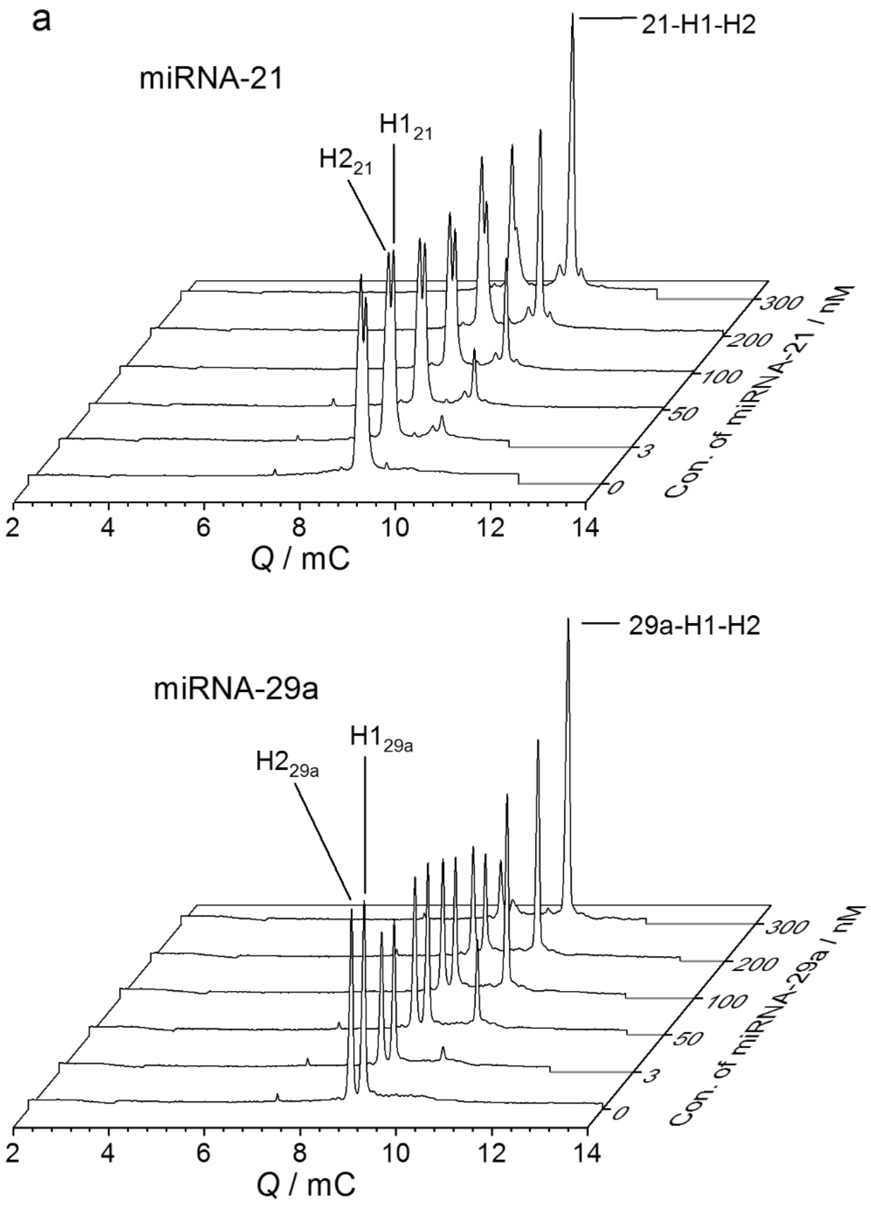
a
miRNA-21
21-H1-H2
H1 21
H2 21
Con. of miRNA-21 / nM
0
3
50
100
200
300
2
4
6
8
10
12
14
Q / mC
29a-H1-H2
miRNA-29a
H1 29a
H2 29a
Con. of miRNA-29a / nM
0
3
50
100
200
300
2
4
6
8
10
12
14
Q / mC

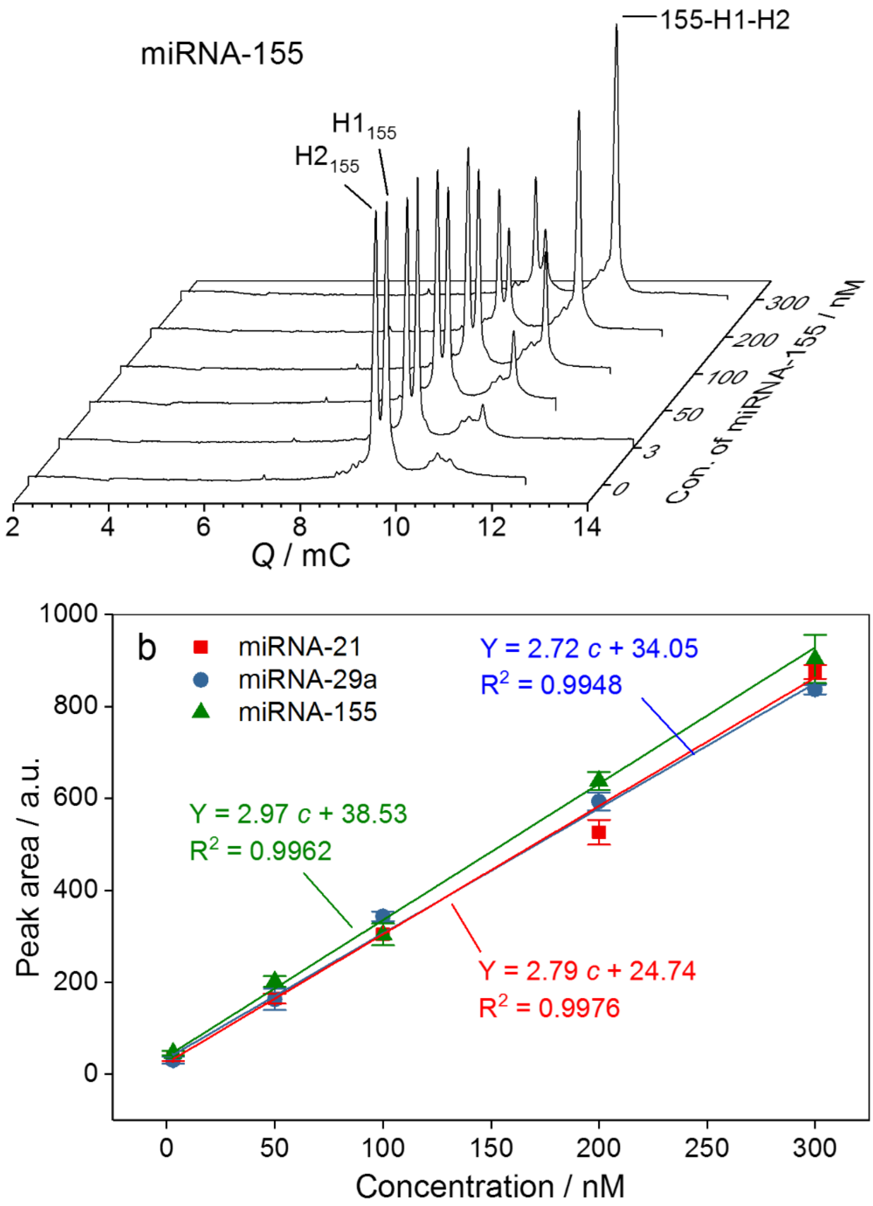

Figure S2 (**a**) *Q*-electropherogram of dsDNA produced by miRNA at the different concentrations; (**b**) The linear relationship of dsDNA product versus the concentration of miRNA. Other conditions as in Figure 2

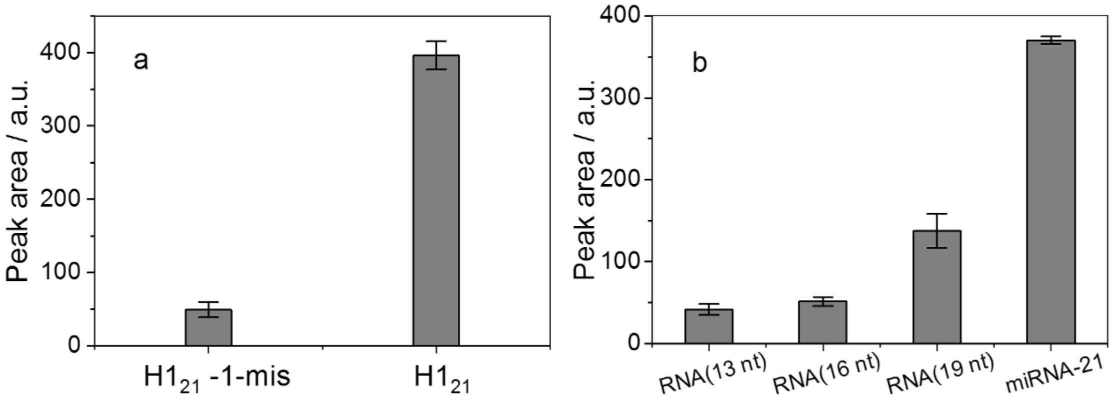

Figure S3 The impact of $H1_{21}$ single base mismatch and various fragments of RNA for the method. (**a**) miRNA-21(100 nM) mixed with their corresponding pairs of $H1_{21}$-1-mis (4 μM) and $H2_{21}$ (6 μM), (**b**) miRNA-21, RNA(13 nt), RNA(16 nt), RNA(19 nt) (100 nM each) mixed with $H1_{21}$ (4 μM) and $H2_{21}$ (6 μM), respectively. And they were incubated at 37 °C for 2.5 h. Other conditions as in Figure 2

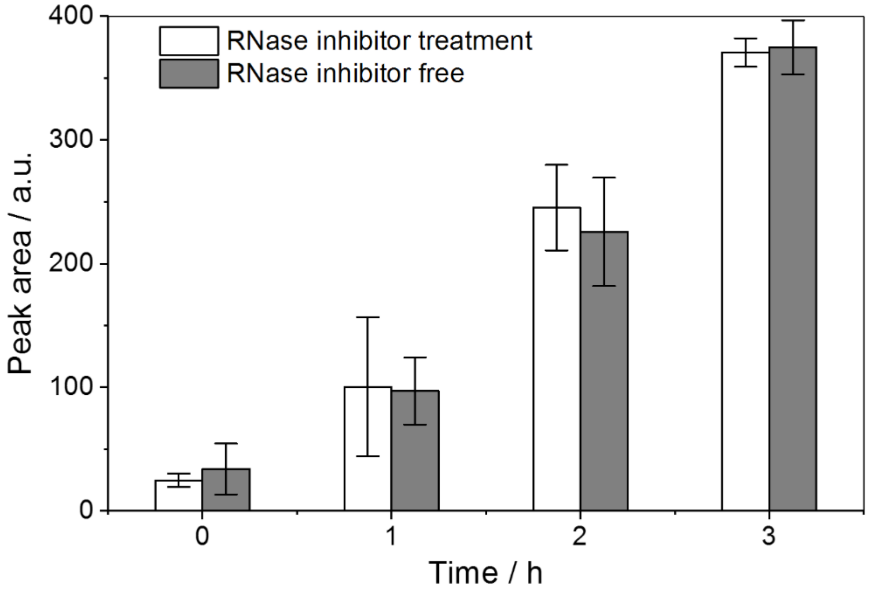

Figure S4 Dynamic monitoring of miRNA stability within effective time by adding RNase inhibitor. Mixture: miRNA-21 (100 nM) added with their corresponding pairs of $H1_{21}$ (4 μM) and $H2_{21}$ (6 μM), RNase inhibitor (20 U), and incubated at 37 °C. Other conditions as in Figure 2

Table S1. Sequences of the oligonucleotides used in the experiments.

Name	Sequences (5′–3′)
miRNA-21	UAG CUU AUC AGA CUG AUG UUG A
miRNA-29a	ACU GAU UUC UUU UGG UGU UCA G
miRNA-155	UUA AUG CUA AUC GUG AUA GGG GU
miRNA-205	UCC UUC AUU CCA CCG GAG UCU G
miRNA-141	UAA CAC UGU CUG GUA AAG AUG G
RNA (19 nt)	UAG CUU AUC AGA CUG AUG A
RNA (16 nt)	UAG CUU AUC AGA CUG A
RNA (13 nt)	UAG CUU AUC AGA C
$H1_{21}$	TCA ACA TCA GTC TGA TAA GCT ACC TAT GTG GAT AGC TTA TCA GAC T
$H2_{21}$	TAA GCT ATC CAC ATA GGT AGC TTA TCA GAC TCC TAT GTG GA
$H1_{29a}$	CTG AAC ACC AAA AGA AAT CAG TCG TCT GTA CTG ATT TCT
$H2_{29a}$	ATC AGT ACA GAC GAC TGA TTT CTC GTC TGT
$H1_{155}$	ACC CCT ATC ACG ATT AGC ATT AAA ACA CGT TAT GGT ACT TTT AAT GCT AAT CGT G
$H2_{155}$	AGC ATT AAA AGT ACC ATA ACG TGT TTT AAT GCT AAT CGT GCA CGT TAT GGT AC
$H1_{21}$-1-mis	TCA AGA TCA GTC TGA TAA GCT ACC TAT GTG GAT AGC TTA TCA GAC T
